# The Lebanese Healthcare Crisis: An Infinite Calamity

**DOI:** 10.7759/cureus.25367

**Published:** 2022-05-26

**Authors:** Mohamad Fleifel, Khaled Abi Farraj

**Affiliations:** 1 Endocrinology, American University of Beirut Medical Center, Beirut, LBN; 2 Biology, American University of Beirut, Beirut, LBN; 3 Medical and Health Sciences, University of Balamand, Beirut, LBN; 4 Endocrinology, Diabetes, and Metabolism, Lebanese American University Medical Center, Beirut, LBN; 5 Internal Medicine, Lebanese American University Medical Center, Beirut, LBN

**Keywords:** healthcare, lebanese crisis, covid-19, beirut port explosion, lebanon

## Abstract

In the past, a number of events rocked Lebanon, a small region of the previously prestigious Phoenician civilization. Whether it was mandates, wars, or economic compromises, the country always seemed to rise up again to a prominent stature in the Middle East. Once known as Switzerland of the East, Lebanon was torn apart by the works of sectarian battles during the civil war from 1975 to 1990. Since then, the country has never been the same with the turmoil left and right. Despite all of that, the healthcare sector has been one of the most prominent in the Middle East and the entire Arab world with accomplished physicians returning from immigration to serve their country. Lebanon excelled in holding first-time international conferences, performing medical interventions, and offering one of the best healthcare education and training to its juniors. The most recent setbacks since late 2019 have, however, held Lebanon back and subsequently handcuffed the healthcare system, leading to the impactful demise of the once glorious care. Nevertheless, the healthcare system remains one of the top-tier domains fighting against the coronavirus disease 2019 (COVID‐19) pandemic and the failings of the rocked state.

## Introduction and background

The red arrow of calamities

**Figure 1:**
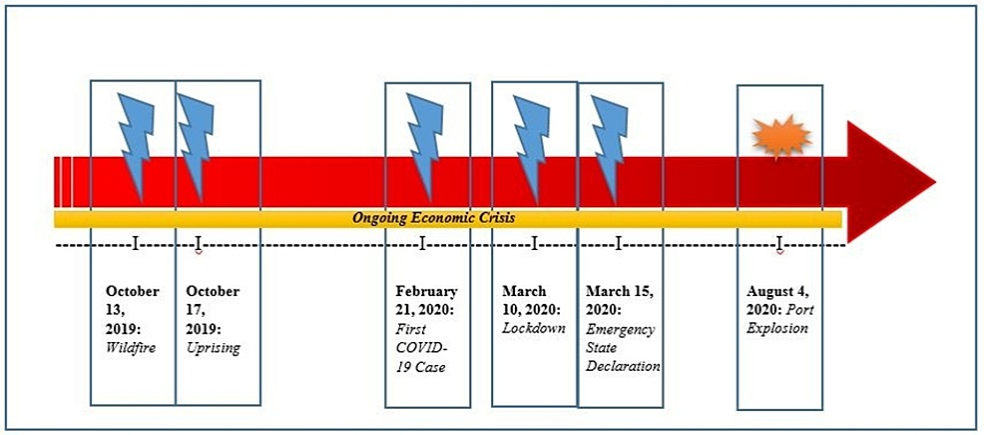
“The Red Arrow of Calamities” represents the major timeline of events in Lebanon from October 13, 2019, to August 4, 2020 The increasing red intensity at the tip of the arrow indicates a continuation of the unfortunate events that Lebanon is still suffering from. ​​​​​Image credit: Created by Dr. Mohamad Fleifel.

As of December 2021, the last 25 months have taken a drastic toll on Lebanon and all of its sectors with healthcare being one of the most notable (Figure 1). It started with the October 2019 Lebanese Wildfire, documented as the worst in 50 years, that encompassed multiple forest areas in Southern and Mount Lebanon. This resulted in more than 50 nonfatal injuries and one death as the fire spread to residential areas [1]. A few days later, a revolutionary uprising was led against the failing governmental system with a collapsing economy. The country was subsequently incapacitated by the batches of confrontations between the armed forces and civilians and also civilians among themselves. Hospital emergency departments (EDs) across the country received numerous injured people, from various sides, with more than 10 individuals losing their lives [2,3]. Concomitantly, at the same point in time as the rest of the world, Lebanon was coping with the wrath of the novel coronavirus (CoV) disease 2019 (COVID‐19).

The first case in the country was reported on February 21, 2020 [4]. Lebanon went into a nationwide lockdown on March 10, 2020, after the first COVID-19-related mortality, and an overall health emergency state declaration was implemented on March 15, 2020 [5]. The different phases of lockdowns negatively impacted the ongoing economic crisis as the country went into further misery with approximately more than 50% of the Lebanese population falling below the poverty line [6]. As of December 2021, Lebanon has recorded over 600,000 confirmed COVID-19 cases with over 8,500 mortalities [4]. On August 4, 2020, many lives endured a gruesome event of irreparable trauma as the unprecedented Beirut port explosion displaced approximately 300,000 individuals and left behind more than 7,000 casualties, including 218 deaths [7]. It was recorded as one of the most seismic artificial nonnuclear explosions ever, with a magnitude of 3.3 ml on the Richter scale [8]. As a result, healthcare centers were extremely burdened with innumerable patients across the hours following the blast resulting in more than 1,000 regular ward and critical care admissions [9].

**Figure 2:**
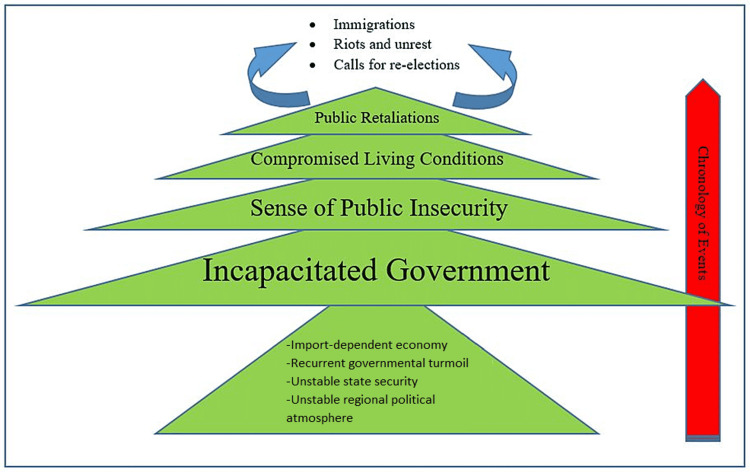
“The Cedargram” represents a blueprint of the recycled escalation of historical events that Lebanon passes through The diagram was created in the form of a cedar tree, the known emblem of the Lebanese flag. It indicates the pyramidal staging of events starting with an accumulation of social, political, and economic dealings in the bark and ending with the resulting retaliation. Image credit: Created by Dr. Mohamad Fleifel.

Currently, Lebanon remains in the depth of political and socioeconomic uncertainties. A tense political state ready to erupt into chaos at any moment, a continuously failing economy, and a relatively staggering COVID-19 morbidity and mortality rates have all left the country’s healthcare system in limbo. As a result, this crisis seems to be infinite as it is resulting in a never-ending snowballing effect, still shaping the lives of many in Lebanon with some choosing to retaliate by face-to-face confrontations with governmental agents, while others prefer to flee in search of a brighter tomorrow (Figure 2). Therefore, the aim of this article is to review the current status of the Lebanese healthcare system in the midst of coincidental and unprecedented national situations based on existing literature, media reports, and subjective insight.

## Review

Methodology

Gathering information about our article's goal was challenging with the scarcity of validated statistical economic data and detailed unbiased evaluation of the political state in accredited journals. Our data was gathered based on: (1) International and local websites that discussed or reported the topics of "healthcare," "immigration of healthcare workers (HCW)," "Beirut port explosion," and "rise in gasoline prices"; (2) medical journals on PubMed and Google Scholar were surfed for specific keywords including "Lebanese crisis," "Beirut port explosion," "Mental health of healthcare workers," "Lebanese healthcare workers," and "Burnout in healthcare," all highlighted the topic of the negative impact of the described situations on the psychosocial state of HCW, and (3) subjective documentation from the authors was intertwined along the article to reflect the dramatic effect of the entire situation.

The exodus of healthcare personnel

There has never been accurate statistical data providing an estimate of the number of medical professionals that left Lebanon since the economic decline and the port blast. An article reported, based on officials from the Lebanese Order of Physicians (LOP), that approximately 1,000 to 15,000 registered doctors have already immigrated to other countries with financially lucrative offers where political and economic conditions are stable [10]. This has been the case for fresh medical graduates as they seek a better future for themselves and their families. American Board-Certified Lebanese doctors have found new homes in the likes of the Arab Gulf and the United States of America (USA), in addition to the francophone doctors that immigrated to France and to other French-speaking European countries. Hospitals have opted to offer contracts with some percentage of “fresh” USD (i.e., cash), nearly a quarter of what these HCW used to receive, in order to attract some personnel to stay. Despite such attempts, the American University Beirut Medical Center (AUBMC), the country’s leading private hospital, documented that nearly 40% of the emergency medical staff have quit for opportunities abroad [10].

The challenging hurdles for the healthcare system

After the Lebanese Ministry of Public Health (MOPH) began lifting subsidies on many drugs across the country, prices started to rise steeply [11]. Many drug prices have unevenly doubled to quadrupled in Lebanese Pounds (LBP), with some individuals not even being able to afford baby food, vitamins, and antipyretics, all of which were previously relatively considered reasonable. This has led people to buy medications and nutritional supplements from neighboring countries like Syria, Jordan, and Turkey, while others opted to receive drugs from relatives and friends abroad from the likes of Europe and the United States. However, a considerable portion of the population does not have either option as they are limited to what their money-strapped state provides. With a current minimal wage of less than 30 USD, such individuals choose to exhaust their monthly salaries to buy necessities, including medications, or reluctantly avoid their costly therapies. Hospital admissions have seen an unprecedented rise in patients presenting to emergency rooms because of clinical manifestations from their inability to afford diuretics, inhalers, antidiabetic drugs, and others [12]. This is of course accompanied by their reluctance to present in the first place because of lack of funds and medical insurance’s strict policies that might prevent hospitals from ordering specific laboratory testing or performing certain management.

The shortage in medical supplies including medications, non-invasive therapy (NIH) machinery, nebulizers, dialysis machines, surgical kits, vital sign monitors, and oxygen tanks has left the healthcare system paralyzed as it attempts to fight against both a pandemic and a fragile economy. Even with the international donations that the country received over the past months, the lack of fuel and electricity renders supplies like NIH useless at homes, thus resulting in exacerbation and decompensation of chronic cardiopulmonary cases. This was mostly evident around the month of August 2021 when the Lebanese Central Bank - Banque du Liban (BDL) - started to show signs of inability to continue subsidizing fuel imports, thus resulting in skyrocketing of the diesel and gasoline prices.

The immediate shortages started to follow, and more hoarding of fuel continued by gas stations. This negatively influenced transportation systems for patients seeking emergent care; ambulances also suffered in some cases as well. Some hospital departments had to cut down the schedule of their medical staff as an alternating “week-on and week-off” policy was implemented to save fuel. A public call was made by three of the major medical centers in the Greater Beirut area as the AUBMC, Rafik Hariri University Hospital (RHUH), and Al Makassed Hospital pleaded for national and international assistance for fuel. Certain hospitals temporarily shut down some of their sections in elective surgery, dialysis units, critical care, and regular wards because of the lack of fuel since many of the major machinery were dependent on electrical power. Senior physicians at RHUH announced that around 10 critical care patients and more than 20 newborns, all ventilator- and incubator-dependent, respectively, were at very high mortality risk as a result of the fuel crisis by the end of August 2021 [13].

Patients have shifted their visits toward the public healthcare sector in terms of a load of emergent and ambulatory care. This has since been due to the relatively lower cost that these medical institutes necessitate, with the likes of RHUH being the leading figure in accepting individuals from all over Lebanon. However, the counter to that has been the already-mentioned lack of supplies that the public sector might suffer from in comparison to private hospitals, in addition to the fast depletion of important resources. This became evident during the spikes in COVID-19 admissions from the fall to winter of 2020.

The psychologically impacted healthcare crew

HCW are sustaining psychological suffering, like everyone else in the country of Lebanon. These providers were the front liners during the Beirut port blast and are still battling the COVID-19 pandemic. A cross-sectional study that measured the mental health effects among a total of 374 participants, including medical students (46.0%) and HCW (54.0%), during these two milestone events documented levels of perceived anxiety, depression, and stress [14]. Using the 10-item Perceived Stress Scale (PSS-10), around 69.0% and 58.1% of medical students and HCW, respectively, had moderate to severe stress. Approximately 46.8% of students and 48.7% of HCW had moderate to severe anxiety and depression based on the four-item Patient Health Questionnaire for Depression and Anxiety (PHQ-4). Sex, monthly income, and work location were all important factors in the HCW's mental health well-being. Compared to men, depression and anxiety were more prevalent in women with a monthly income of less than four million LBP and in those traveling in between the districts [14].

In addition to COVID-19 and the port explosion, HCW have to deal with the burden of scarce medications, depleting fuel, and the psychosocial aspect of trying to optimize a living for themselves and their families. A sense of demotivation, fatigue, and burnout, if reached, can jeopardize the patients’ safety. The impact of burnout is demonstrated through cynicism, poor job satisfaction, and a decrease in compliance to job tasks, which can further create a tense work atmosphere that can affect colleagues as well [15]. Suboptimal patient care and consequential medical errors have been reported in physician burnout, which resulted in poorer patient satisfaction and subsequent malpractice suits with financial caregivers and hospital costs [15-17]. Social and self-neglect have been previously reported among resident physicians when a high level of fatigue and burnout are reached, with some neglecting social and personal care [18].

The consequences of the Beirut port blast

**Figure 3:**
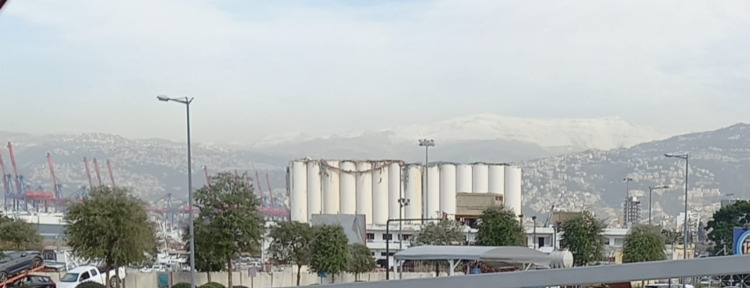
The damaged grain silos (white) located at Beirut port Image credit: Photo taken by the authors on January 12, 2022.

**Figure 4:**
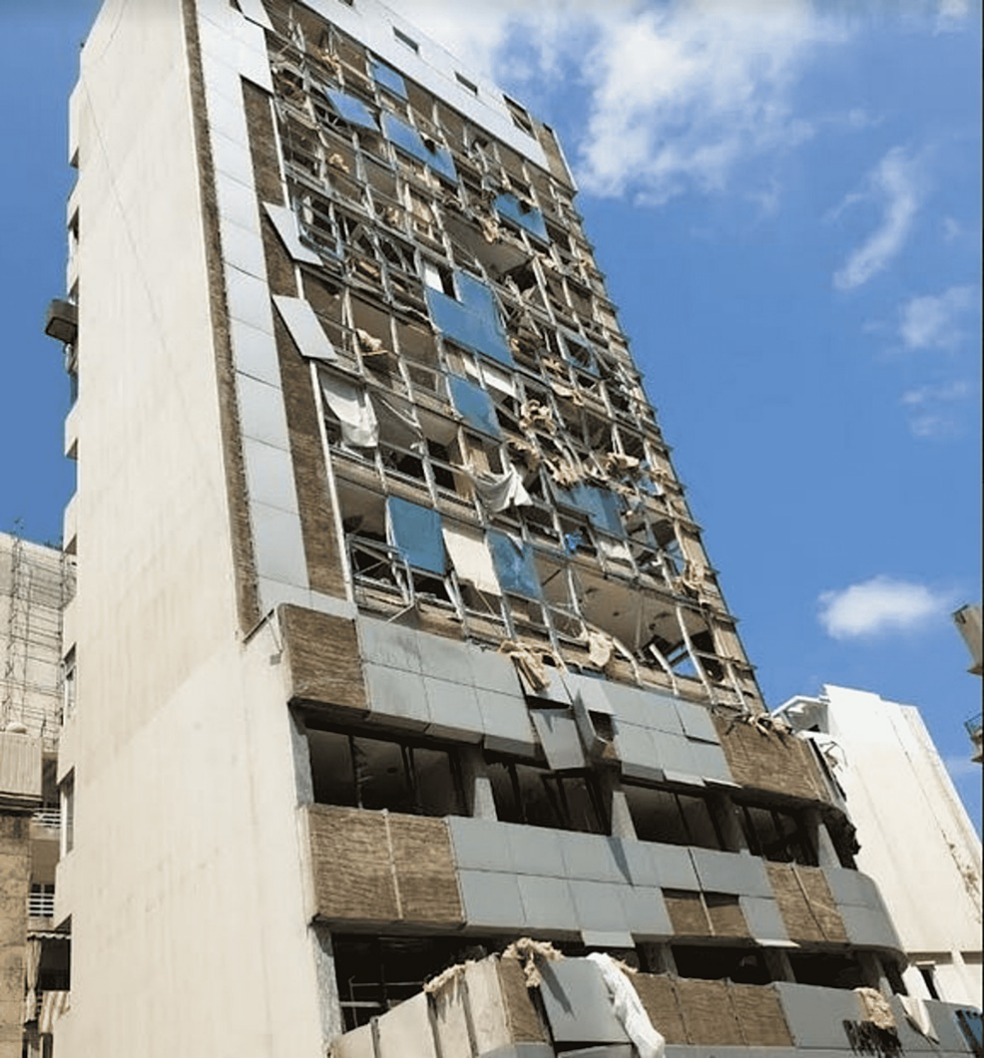
A demolished building near the site of the port explosion Image credit: Photo taken by the authors on August 05, 2020.

**Figure 5:**
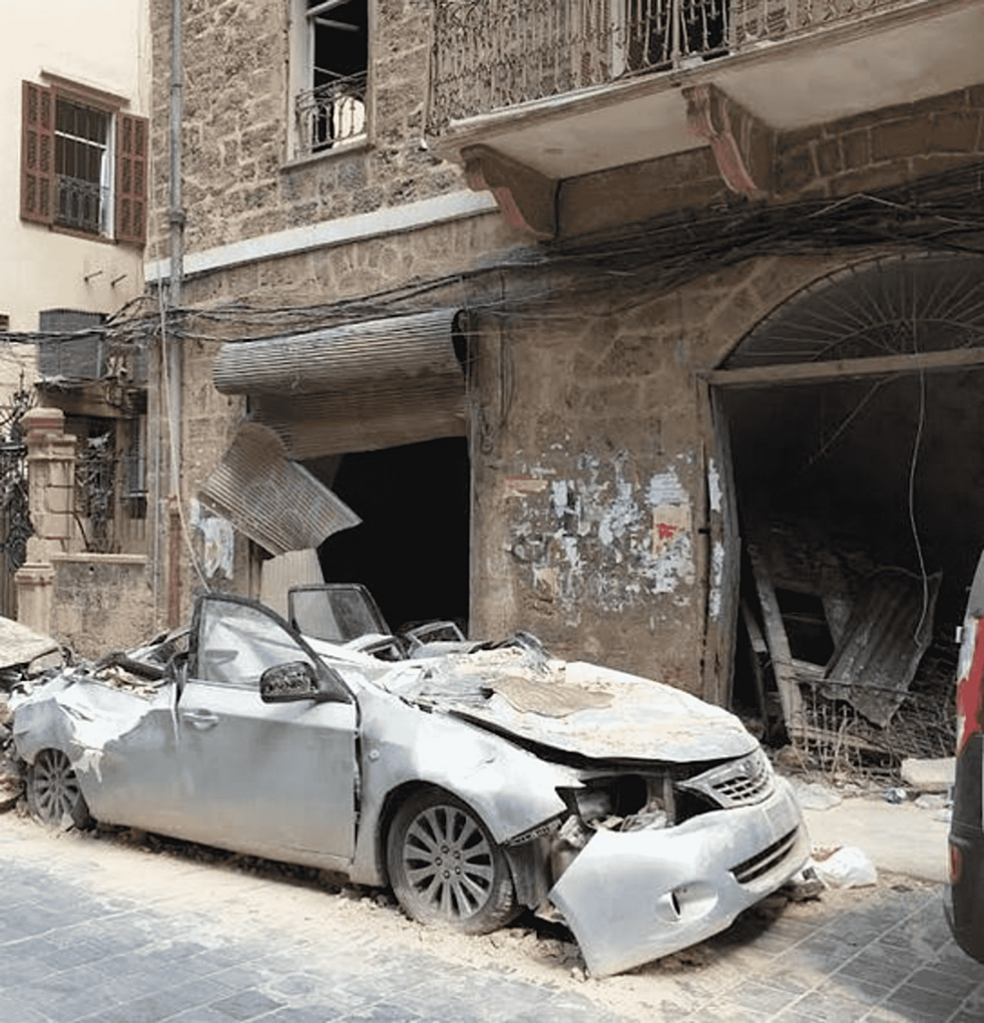
"Rendered into ashes" as the port blast reached the streets of Beirut Image credit: Photo taken by the authors on August 05, 2020.

Lebanon set the record for having the second-largest urban explosion since Hiroshima and Nagasaki on August 4, 2020, after the capital’s port blast incident (Figures 3-5). The authorities seem to agree that the blast was due to a fire from a combustible shipment of ammonium nitrate; however, national and international investigations are still to deduce what triggered the fire and how it could have been avoided. The explosion incapacitated multiple hospitals in its radius and extensively damaged two major centers in Saint George Hospital University Medical Center (SGHUMC) and Lebanese Hospital Geitaoui (LHG).

**Figure 6:**
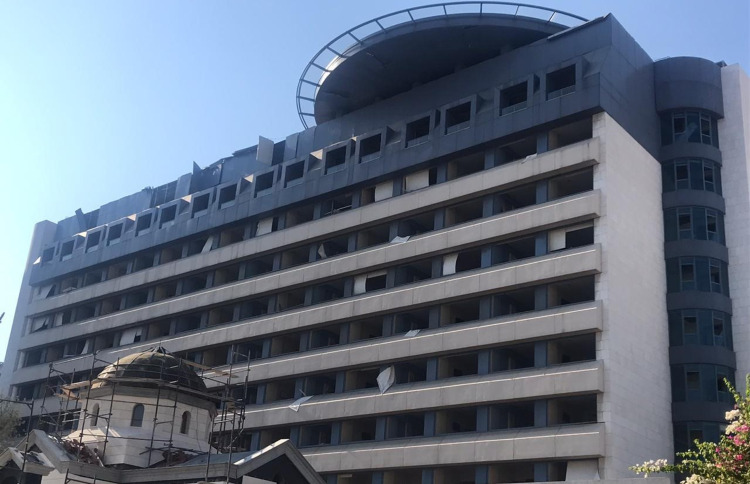
The damaged exterior of SGHUMC Image credit: Photo taken by the authors on August 05, 2020. SGHUMC: Saint George Hospital University Medical Center.

**Figure 7:**
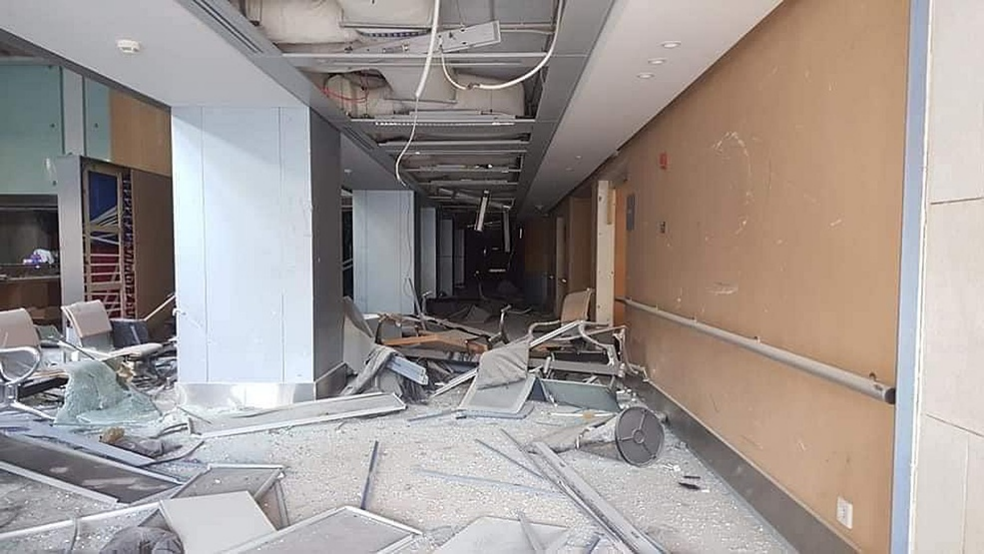
The destroyed hallways of SGHUMC Image credit: Photo taken by the authors on August 05, 2020. SGHUMC: Saint George Hospital University Medical Center.

**Figure 8:**
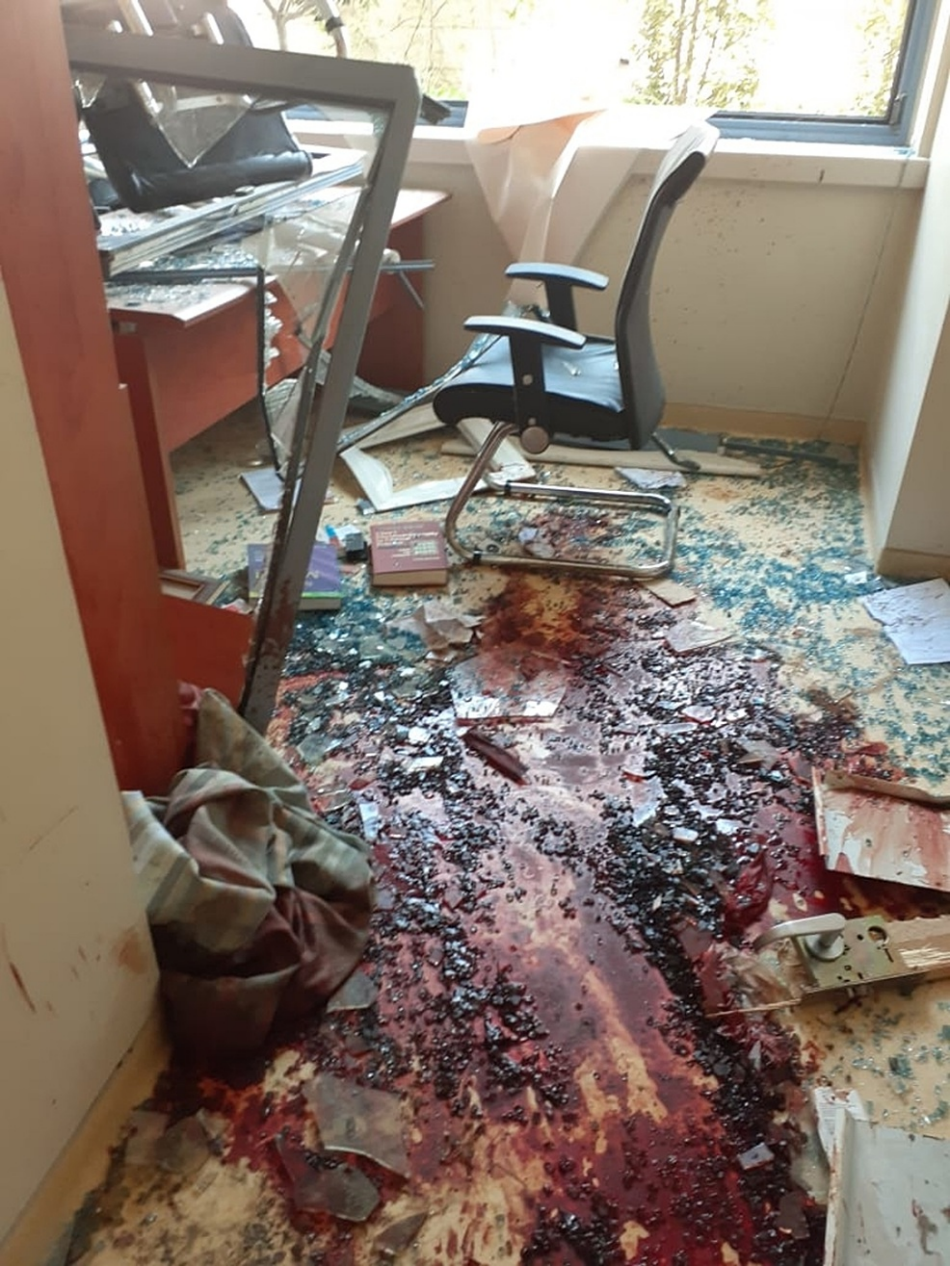
Total destruction of a clinic at SGHUMC in the aftermath of the port blast Image credit: Photo taken by the authors on August 05, 2020. SGHUMC: Saint George Hospital University Medical Center.

SGHUMC became non-operational for the first time in 142 years on that evening with the lack of electrical power and the vast destruction in outpatient and inpatient facilities (Figures 6-8; beware of graphic content). HCW resorted to treating any incoming blast victims on the pavement outside the ED and in the parking lot while attempting to make the best out of what was left of functioning medical equipment and medications [19,20]. The blast killed four registered nurses at the institute and injured multiple HCW and patients. According to an interview with the International Committee of the Red Cross, Beirut-based medical institutes consumed approximately two months’ worth of supplies on the evening of the blast [21]. This resulted in a further dearth of some equipment and drugs at multiple hospitals, crippling both patients’ needs and HCW abilities to provide the best quality of care. Humanitarian needs increased further as the multiple calamities of the explosion, pandemic, and economic collapse altogether compoundedly affected many of the population. Financially able HCW and patients opted to leave the country, on a permanent or temporary basis, as they searched for a better quality of life, overall. 

An everlasting hope

The resiliency of the Lebanese people is a well-known badge of honor. With the self-recycling history of Lebanon, the willpower of the population has been a model for every nation fighting against a failing government. The malleable nature of the Lebanese healthcare system has so far stood the test of time and managed to overcome multiple hurdles. The country has always benefited from the minds of great physicians that chose to return to their homeland and rehabilitate healthcare.

Lebanon was the first country in the Arabian and Middle Eastern regions to introduce percutaneous mitral commissurotomy, MitraClip intervention, intracoronary and carotid stentings, and transcatheter aortic valve implantation [22]. The Lebanese American University Medical Center-Rizk Hospital (LAUMC-RH) was the first institute in the Middle East to introduce the Comprehensive Stroke Center in 2018 [23]. AUBMC was also the first hospital to establish a clinical endocrinology fellowship program in the Middle East [24]. The renowned Middle East Medical Assembly (MEMA), a Beirut-based annual international medical conference that gathers the minds of worldwide physicians to present the latest updates, guidelines, and educational opportunities to its attendees, has been a near-constant congress at AUBMC [25]. A glimmer of hope always resides as long as there is trust in a healthcare system that sustains its resiliency in the face of various hurdles.

## Conclusions

Among the entirety of the comorbid nature of the crises that surround Lebanon, fear resides in the hearts of the population. The likes of Greece, Cyprus, and Venezuela have had their share of economic collapse in previous years; however, none had to combat the additional catastrophes that Lebanon currently has. The perseverance of the population to survive in the midst of wars and various crises is what keeps the people driven to endure and fend off the shadows of further failure. An urgent radical governmental reform is a first step to revive life in the country, which would boost the healthcare system if enough economic and political stabilities are achieved. As previously indicated, the healthcare system has always had the blueprint of success laid out and ready to implement if permitted by the surrounding setting. We hope that our article drives scholars and healthcare executives to continue supporting their system and initiate emergency plans for catastrophic crises that might come ahead.
